# Orthogonal Optimal Design of Multiple Parameters of a Magnetically Controlled Capsule Robot

**DOI:** 10.3390/mi12070802

**Published:** 2021-07-06

**Authors:** Puhua Tang, Liang Liang, Zhiming Guo, Yu Liu, Guanyu Hu

**Affiliations:** College of Electromechanical Engineering, Changsha University, Changsha 410022, China; tphtom@126.com (P.T.); zmguo@ccsu.edu.cn (Z.G.); liuyu@ccsu.edu.cn (Y.L.); hgy197301@126.com (G.H.)

**Keywords:** capsule robot, orthogonal design, CFD (computational fluid dynamics), PIV (particle image velocimetry), operating performance indicators

## Abstract

Magnetically controlled capsule robots are predominantly used in the diagnosis and treatment of the human gastrointestinal tract. In this study, based on the permanent magnet method, magnetic driving and fluid measurement systems for in-pipe capsule robots were established. Using computational fluid dynamics (CFD) and particle image velocimetry (PIV), the fluid velocity and vorticity in the pipe of the capsule robot were calculated and measured. The running characteristics of the capsule robot were numerically analyzed in the curved pipe and the peristaltic flow. Furthermore, the range and variance method of orthogonal design was used to analyze the influence of four typical parameters (namely, pipe diameter, robotic translational speed, robotic rotational speed, and fluid viscosity) on the three operating performance indicators of the capsule robot (namely, the forward resistance of the robot, fluid turbulent intensity near the robot, and maximum fluid pressure to the pipe wall). In this paper, the relative magnitude and significance of the influence of each typical parameter on different performance indicators of the robot are presented. According to the different performance requirements of the robot, the different four parameter combinations are optimized. It is hoped that this work provides a reference for the selection of the appropriate mucus, translational speed, and rotational speed of the robot when it is working in pipes with different diameters.

## 1. Introduction

A capsule endoscopy (i.e., using a capsule robot) is also known as a capsule gastrointestinal endoscopy system or medical wireless endoscopy. In this procedure, patients swallow capsules that have a built-in camera and signal transmission device. These capsules move in the digestive tract with the help of gastrointestinal peristalsis or an external driving field. Doctors use an external image recorder and image workstation to gain knowledge about patients’ digestive tracts, and thus make disease diagnoses. Compared with plug-in gastrointestinal endoscopy, the advantages of capsule endoscopy are that it is painless, noninvasive, safe, and convenient. However, during passive capsule endoscopy, the speed of travel through the esophagus and the movement in the stomach cannot be controlled, thus preventing a complete examination of the GI tract. Heavy, expensive, and complex magnetic navigation systems have been developed to control the capsule movement in the stomach. More recently, a smaller magnetic guidance system using a hand-held magnet has been developed [1]. In 2020, the Jinshan company of China released the world’s first magnetically controlled capsule endoscopy for gastrointestinal examination [2]. The conventional active capsule robot has a smooth surface and cylindrical shape [3]. To improve the propulsive force of the capsule robot in an intestine filled with mucus, the researchers improved the structure of the capsule robot by using a spiral structure on its surface [4,5]. To allow the capsule to be fixed in place in the intestine, some capsule robots have legs or claws on their surface [6,7]. Researchers have also used wheels on the surface of the capsule robot for sampling [8].

At present, many drive modes of capsule robots exist. One method is the internal drive mode, which uses batteries or cables [9,10]; however, this approach has a number of problems, such as insufficient energy supply or inconvenient movement. Therefore, another widely used method is the external drive mode. The drive modes of an external magnetic field are divided into the coil drive mode and the permanent magnet drive mode. In the coil drive mode, multiple groups of electrified coils (mainly Holmhertz coils) are used to produce a uniform magnetic field to drive the magnetic capsule to move in a pipe [11,12]. In this mode, the magnetic field is relatively uniform, but the equipment and operation are relatively complex, and it is difficult to achieve stable and accurate control of the capsule robot. In the permanent magnet drive mode, the magnetic capsule robot is driven to follow the motion in the pipe in the same manner as the linear or rotary motion of the external permanent magnet [13,14]. This mode is relatively convenient to control and operate; however, it is also difficult to control the force balance, and the movement suffers from a slight lag relative to the movement of the external permanent magnet.

When a capsule robot is working in a pipe filled with mucus, many parameters affect its operating performance, which can be mainly divided into the following categories. One category relates to the parameters of the robot, such as robotic structural and operating parameters. Ye et al. used a square magnet to drive the magnetic capsule of the outer spiral structure for translational and rotational motion, and experimentally analyzed the translational speed of the robot at different robotic rotational speeds under different distances between the permanent magnets and capsule robots [15]. Guo et al. applied the Hertz contact theory, finite element method, and experimental means to study the pressure of the capsule robot on the pipe wall at different translational speeds [16]. Zhou et al. used the computational fluid dynamics (CFD) method to calculate the translational speed and resistance torque of external spiral capsule robots with different thread shapes, line numbers, and lead [17]. Wang et al. used finite element software to calculate and experimentally measure the fluid velocity at the pipe outlet under different thread parameters of an external spiral robot [18]. Zhang et al. experimentally studied the translational speed and resistance torque of capsule robots with different thread structures under different oil film thickness [19]. The second category relates to the parameters of the magnetic field, such as intensity and frequency. Nguyen et al. theoretically analyzed, numerically calculated, and experimentally measured the locomotion force of a capsule robot under different gradient fields [20]. Hoang et al. theoretically and experimentally studied the relationship between the magnetic field intensity, and the driving force and torque of an outer spiral capsule robot [21]. The third category relates to the parameters of the working pipe, such as its diameter, shape, inner surface morphology, and material characteristics. Tang et al. calculated the force of a capsule robot in pipes with different diameters, and measured the fluid velocity in the pipe [22]. Zhang et al. experimentally measured the effects of various micro patterns on the friction and mucus adhesion between the robot and the intestine [23]. The final category relates to the parameters of the fluid in the pipe, such as viscosity, density, phase number, and composition of the mucus. Liang et al. used the CFD method to calculate the operating characteristics of spiral capsule robots in the environments of solid–liquid mixed fluid and liquid–liquid mixed fluid [24].

In conclusion, regarding the operating performance of in-pipe capsule robots, the existing research mainly focuses on the influences and optimization of the various parameters. Theoretical research includes contact mechanics and the theory of the lubrication between the capsule robot and intestine, whereas numerical research mainly involves the application of the CFD method. Experimental research focuses on the spiral structural parameters and operating parameters of capsule robots, magnetic field parameters, etc. However, the relative degree and significance of the influence of various parameters on the operating performance of capsule robots has not been discussed.

Using a capsule robot with a permanent drive, in the current study a self-designed measurement system for the fluid flow field was used to verify the accuracy of the proposed calculation method. Furthermore, four different typical parameters (namely, pipe diameter, robotic translational speed, robotic rotational speed, and fluid viscosity) that affect the operating performance of the capsule robot were taken as influencing factors, and the forward resistance of the robot (reflecting the robot’s passing capacity), fluid turbulent intensity near the robot (reflecting the robot’s operating stability), and maximum fluid pressure on the pipe wall (reflecting the degree of damage to the pipe wall) were taken as the optimization objectives. The orthogonal design method was used to analyze the relative degree and significance of the influence of the four parameters on the operating performance of the capsule robot, and the optimal combinations of parameters under different pipe diameters were calculated. This paper provides a basis for the reasonable selection of operating and mucus parameters of capsule robots in pipes of different sizes.

## 2. Working Principle and Related Parameters of Capsule Robot

**Figure 1:**
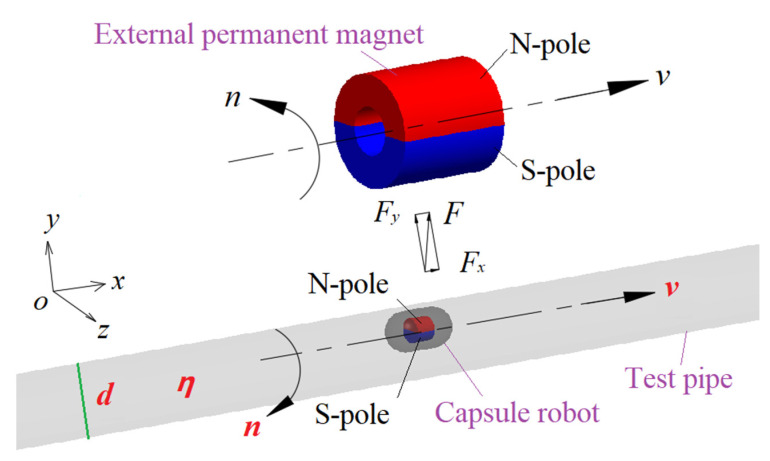
Structure and related parameters of the capsule robot.

In the permanent magnet drive mode, as shown in Figure 1, the external permanent magnet is an annular cylinder, and the internal magnet is a solid cylinder, which is placed in the center position of the robot. The two magnets are separated by a suitable distance. The magnets are diametrically magnetized, and one half are N-pole (red semi-annular cylinder), and the other half are S-pole (blue semi-annular cylinder).

The external permanent magnet is driven by a motion platform to make a translating and rotating (i.e., precessing) motion along the *x*-axial direction. Due to the magnetic force of the external permanent magnet, the capsule robot is driven to make a slightly delayed precessing motion along the *x*-axis. The translational velocity of the capsule robot along the *x*-axis is the same as that of the external permanent magnet in magnitude and direction, and the rotational speed of the capsule robot around the *x*-axis is the same as that of the external permanent magnet, but their rotational direction is opposite. The force of the robot in the *x*-axial direction includes the *x* component of the magnetic force, the mucus resistance, and the friction force with the pipe wall. The main purpose of the rotation of the capsule robot is to reduce the friction resistance between the robot and the pipe wall, and improve the forward driving force of the robot. The main parameters that affect the operating performance of the capsule robot are pipe diameter *d*, robotic translational speed *v*, robotic rotational speed *n*, and fluid dynamic viscosity *η*.

## 3. Numerical Calculation Method

### 3.1. Mathematical Model of Numerical Calculation

In CFD, namely, computational fluid dynamics, the integral and differential terms in the governing equations of fluid mechanics are approximately represented as discrete algebraic equations, and these equations are solved by computer to obtain the numerical solutions at discrete points in time and space.

When the robot is working in a pipe filled with fluid, the fluid is assumed to be incompressible and unaffected by temperature. The fluid also satisfies the following conservation equations [25]:

(1) $$\frac{\partial \rho }{\partial t}+\nabla \cdot (\rho u)=0$$

(2) $$\frac{\partial (\rho u_{h})}{\partial t}+\nabla \cdot (\rho u_{h}u)=-\frac{\partial p}{\partial h}+\frac{\partial \tau_{xh}}{\partial x}+\frac{\partial \tau_{yh}}{\partial y}+\frac{\partial \tau_{zh}}{\partial z}+f_{h}\;(h=\;x,\;y,\;z)$$

where *ρ* is fluid density; $\nabla =i\frac{\partial }{\partial x}+j\frac{\partial }{\partial y}+k\frac{\partial }{\partial z}$, ***i***, ***j***, and ***k*** are unit vectors of *x*, *y*, and *z* axes, respectively; *u_h_* (*h* = *x*, *y*, *z*) is the component of velocity vector ***u*** in *x*, *y*, and *z* axes; *p* is pressure; *τ_xh_*, *τ_yh_*, and *τ_zh_* are components of molecular viscous stress *τ*; and *f_h_* is body force, if the force is gravity and its direction is negative along the *y* axis, then *f_x_* = 0, *f_y_* = −*ρ*g, *f_z_* = 0.

Equations (1) and (2) are the dynamics equations of a viscous fluid. Using the CFD method to calculate the above equations, the fluid field near the robot can be obtained, and the force of the fluid on the robot can also be obtained. The solving process of the CFD method used in this paper includes: system modeling, grid division, boundary conditions and parameter setting, and numerical solution.

### 3.2. Fluid Turbulent Intensity

When the robot precesses in a pipe filled with fluid, the fluid near the robot not only flows along the axial direction of the pipe, but also moves in the tangential direction, so the fluid flow is turbulent. At this time, the fluid pressure and velocity are changed with time. Turbulent intensity is the most important characteristic quantity to describe the turbulent motion characteristics of fluid, and is the relative index used to measure the turbulence. Turbulent intensity, broadly defined, is the ratio of the mean square root of the pulsating velocity to average velocity. The greater the turbulent intensity of the fluid near the robot, the more disordered the relative motion of the fluid, and the worse the stability of the robot.

(3) $$I_{t}=\frac{v^{\prime }}{\bar{v}}=\frac{\sqrt{\frac{1}{N}\sum_{i=1}^{N}{\left(v_{i}-\bar{v}\right)}^{2}}}{\bar{v}}$$

where *I_t_* is turbulent intensity, *v*′ is mean square root of the pulsating velocity, $\bar{v}$ is average velocity, and *N* is number of sampling points in a period of time.

### 3.3. System Modeling

**Figure 2:**
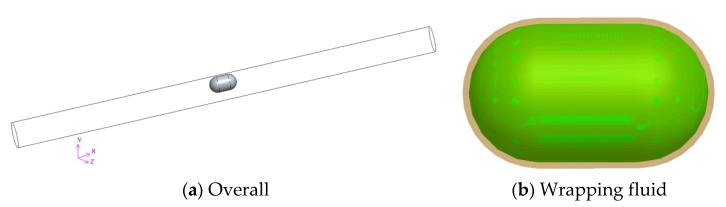
Geometric model of the robot system (*d* = 18 mm).

As shown in Figure 2, the whole system included a capsule robot, a pipe, and fluid. The robotic outer diameter was 10 mm, its length was 18 mm, both of its ends were hemispherical, and its middle part was cylindrical. With reference to the diameter of the human small intestine, the pipe diameter was set to 18 mm and its length was 300 mm. The fluid in the pipe was selected to be silicone oil, as it is equivalent to intestinal mucus for endoscopy examination. The mixture of simethicone powder tablets and drinking water can remove air bubbles and clean the intestines [26].

Considering the translational and rotational motion of the capsule robot, to refine the grid of the fluid zone around the capsule robot and analyze the influence of the fluid characteristics of this zone on the operation of the robot, a layer of wrapping fluid was added around the robot. As shown in the yellow zone of Figure 2b, the shape of the wrapping fluid was the same as that of the capsule robot, and its thickness was 0.5 mm. To satisfy the numerical calculation, the distance between the wrapping fluid and the upper wall of the pipe was 0.5 mm.

### 3.4. Grid Division

**Figure 3:**
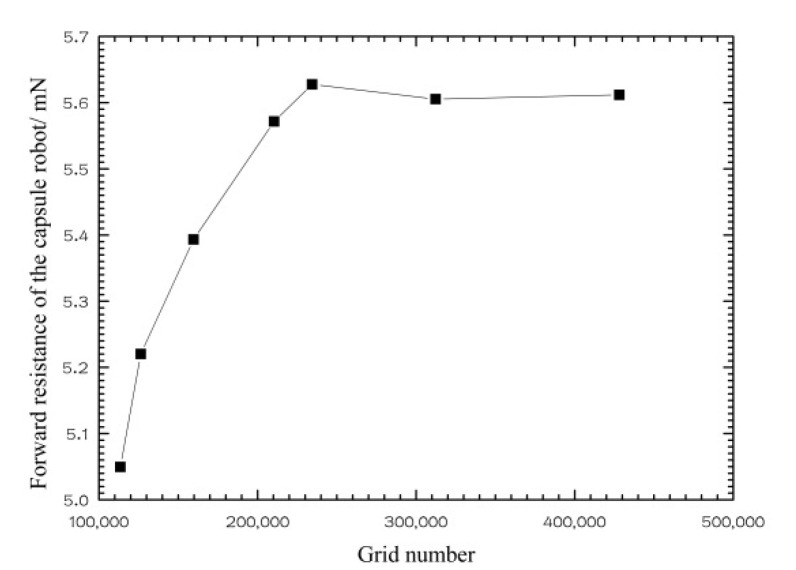
Grid independence analysis diagram (*d* = 18 mm).

**Figure 4:**
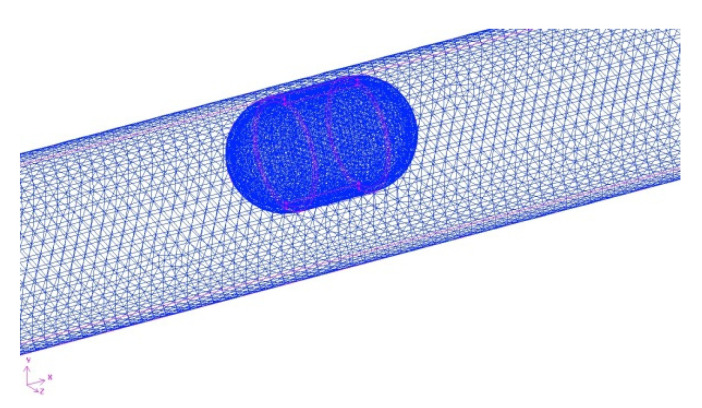
Grid diagram of the robot system (*d* = 18 mm).

The fluid in the pipe includes two fluid zones: the wrapping fluid around the robot and the residual fluid in the pipe. Unstructured tetrahedral meshes are used in the two fluid zones, and refined meshes are used in the wrapping fluid zone. Under the condition that the quality of the grid meets the requirements of the calculation, the number of grids is increased, and the time step size is decreased until the numerical results tend to be stable. Figure 3 shows the relationship between the grid number of the system and forward resistance of the robot. When the grid number of the system was more than 230,000, the forward resistance of the robot tended to be steady. After comprehensive consideration, the grid number of the wrapping fluid zone was 17,300, the grid number of the remaining fluid zone was 234,464, the total grid number is 251,764, and the time step size was selected to be 0.0005 s. The local grid of the fluid around the robot is shown in Figure 4.

### 3.5. Boundary Condition and Parameter Setting

The standard *k*-*ε* model was adopted for the turbulent model, and the standard wall function was used near the pipe wall. According to the actual application, the translational speed *v* of the capsule robot ranged from 0.02 to 0.06 m/s, the rotational speed *n* of the capsule robot ranged from 60 to 180 r/min, the variation range of the pipe diameter *d* was 14~22 mm, and the variation range of fluid viscosity *η* was 0.005~0.1 Pa·s. The sliding mesh and dynamic mesh technology were used to handle the rotation and translation of the robot, and the robot was assumed to precess along the *x*-axis.

According to the characteristics of intestinal tract, both ends of the pipe were treated with walls, and it was assumed that the fluid in the pipe does not flow initially. The initial values of all zones were set to zero, and the calculation convergence precision of each variable was 0.001.

## 4. Experimental Measurement

### 4.1. Measurement System

**Figure 5:**
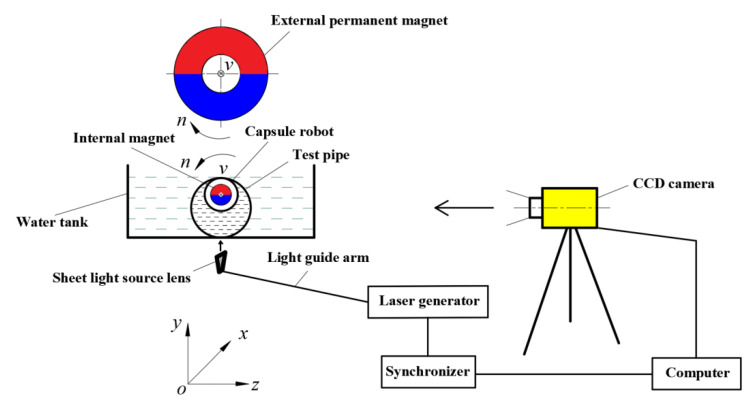
Measurement principle diagram of fluid flow field in the pipe of the capsule robot.

To verify the CFD method used in this paper, particle image velocimetry (PIV) technology was used to measure the fluid flow field. As shown in Figure 5, a computer and synchronizer were used to control a charge coupled device (CCD) camera to take pictures, and the laser generator was used to synchronously generate a pulse laser. The thickness of the pulse laser was 1 mm, which is used to illuminate the fluid region to be measured (i.e., the *xoy* cross section passing through the robot center) in the pipe, so that the CCD camera was able to continuously capture the image of the tracer particles in this plane. After data processing, the velocity field of the fluid at the tracer particles could be solved; that is, the fluid field of the *xoy* plane near the capsule robot was also obtained. The tracer particles were hollow glass beads with density of 1.05 g/cm^3^ and diameter of 8–12 μm, and have good flow followability.

**Figure 6:**
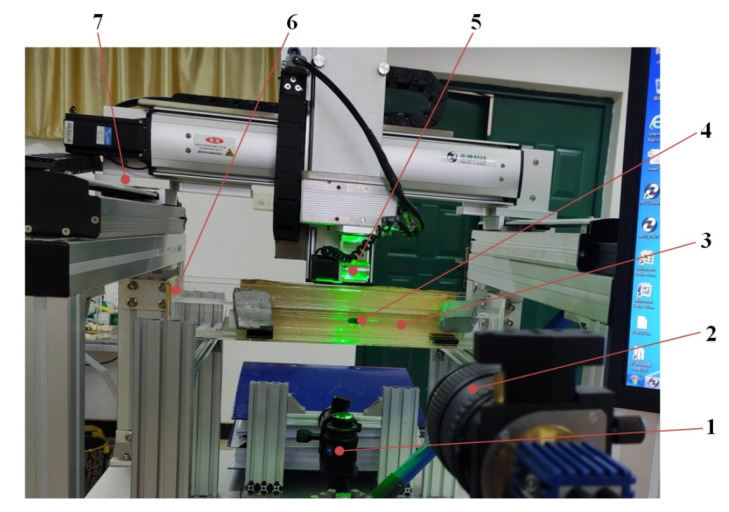
Experimental system for measuring the fluid flow field in the pipe. 1—Sheet light source lens; 2—CCD camera; 3—Experimental glass pipe; 4—Capsule robot; 5—External permanent magnet; 6—Square water tank; 7—Multi-axial motion platform.

According to the working principle shown in Figure 5, an experimental system for measuring the fluid flow field of magnetically controlled in-pipe robots was designed and manufactured. As shown in Figure 6, the experimental system was composed of the driving module of the robot and the measurement module of the fluid field. The driving module included a multi-axial motion platform, external permanent magnet, capsule robot, test pipe, rotating motor, motion control card, and computer. The measurement module included the PIV system, support, water tank, pressure block, and gasket. The main components of the PIV system were the pulse laser generator, sheet light source lens, CCD camera, synchronizer, image analysis system, and computer.

**Figure 7:**
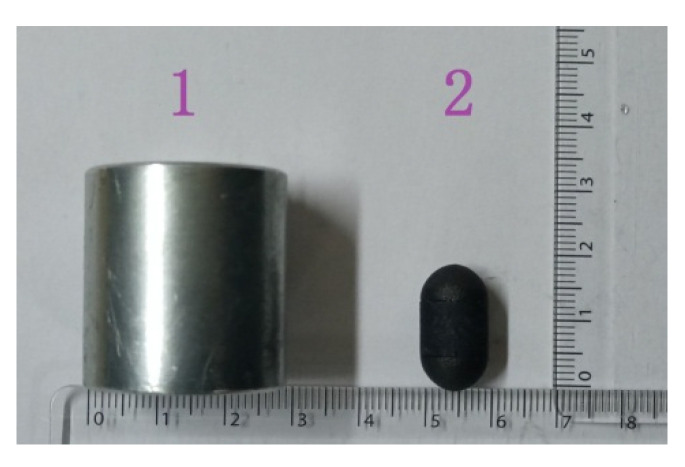
External permanent magnet and capsule robot. 1—External permanent magnet; 2—Capsule robot.

**Figure 8:**
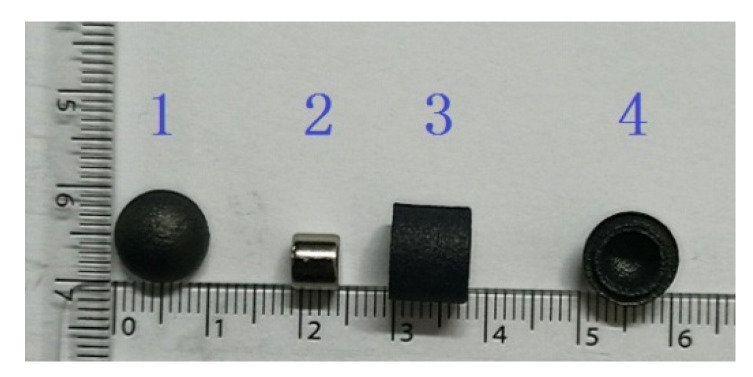
Parts of the capsule robot. 1—Front end cap; 2—Magnet; 3—Middle section; 4—Rear end cap.

Figure 7 shows the external permanent magnet and capsule robot. The external permanent magnet was an annular cylinder with an outer diameter of 28 mm, an inner diameter of 12 mm, and a length of 30 mm. As shown in Figure 8, the capsule robot has an internal magnet, which is a solid cylinder, with a diameter of 6 mm and length of 5 mm. The shell of the capsule robot was black and was manufactured from bioplastic. The material of the external and internal magnet was N38 NdFeB. To ensure the normal operation of the capsule robot in pipes of different sizes, the distance between the external magnet and the internal magnet remained unchanged.

### 4.2. Comparison between Numerical Calculation and Experimental Measurement

**Figure 9:**
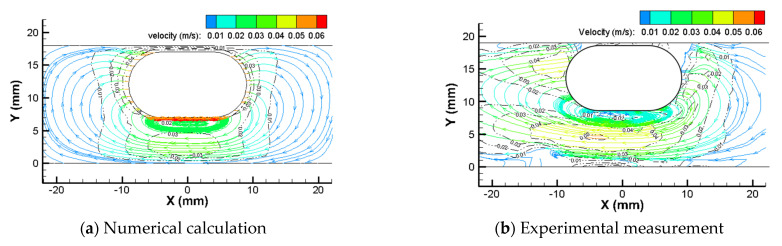
Streamlines and velocity of fluid around the capsule robot (*d* = 18 mm, *v* = 0.05 m/s, *n* = 60 r/min, and *η* = 0.02 Pa·s).

Figure 9 shows the streamline and velocity distribution of fluid in the pipe at the *xoy* plane through the center of the capsule robot when the robot precesses in a pipe with a diameter of 18 mm filled with silicone oil with dynamic viscosity of 0.02 Pa·s, at the robotic translational speed *v* of 0.05 m/s and robotic rotational speed *n* of 60 r/min. The zero point of the *x*-axial coordinate passes through the center of capsule, and the zero point of the *y*-axial coordinate passes through the bottom of pipe. In the experiment, the pipe diameter shown is slightly larger than 18 mm, which is due to the refraction of light passing through the glass pipe.

The trajectory of the fluid streamline around the capsule robot is a circular line from the head of the capsule to the end of the capsule. A large fluid vortex is formed at the bottom of the robot, and the fluid velocity is larger in the middle of the region between the capsule and the pipe. The experimental results show that the overall distribution of fluid streamline around the capsule robot is basically the same as the numerical results, but the streamline in a few regions is slightly chaotic and the fluid velocity is slightly different. This is as, in the experiment, the capsule robot precesses in a slight swing mode.

Vorticity is usually used to measure the magnitude and direction of a vortex, and is defined as the curl of the fluid velocity vector, with unit s^−1^. If a vorticity source exists, vortices of different sizes will be produced. The direction of the vorticity is determined by the right hand rule. When the right hand clenches the fist, the direction of four fingers is the fluid rotational direction, and the thumb points to the direction of vorticity [27].

The larger the vorticity of the fluid around the capsule robot, the greater the fluid rotational intensity, the more chaotic the fluid flow field, the greater the fluid turbulent intensity near the robot, and the worse the stability of the robot. The vorticity is calculated as follows.

(4) Ω=2ω=∇×U

where Ω represents vorticity; ***U*** represents linear velocity; and ***ω*** represents rotational angular velocity.

**Figure 10:**
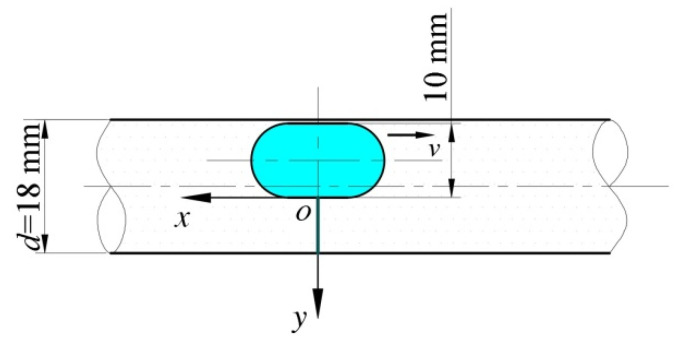
Coordinate system for comparison of the numerical calculation values and experimental measurement values.

**Figure 11:**
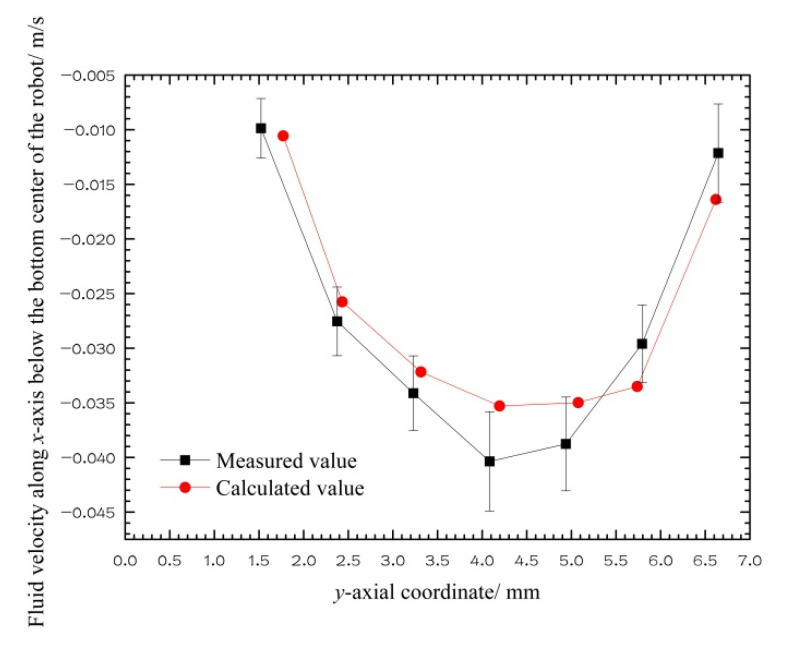
*x*-axial velocity of fluid below the bottom center of the robot (*d* = 18 mm, *v* = 0.05 m/s, *n* = 60 r/min, and *η* = 0.02 Pa·s).

To quantitatively compare the calculated values with the measured values, as shown in Figure 10, a new coordinate system was set up with the bottom center of the capsule robot as the coordinate origin. The *x*-axial positive direction is horizontal to the left, and the *y*-axial positive direction is vertical to the down. Figure 11 is a comparison diagram of the CFD calculation and PIV measurement values of the *x*-axial component *u_x_* of the fluid velocity on the *y*-axis below the capsule robot in the coordinate system shown in Figure 10. The figure shows that the direction of the velocity *u_x_* is to the left, which is opposite to the direction of the robotic translational velocity. Its value first increases, then decreases from the bottom center of the robot to the pipe bottom. The change trend and numerical value of CFD calculation and PIV measurement results are consistent.

**Figure 12:**
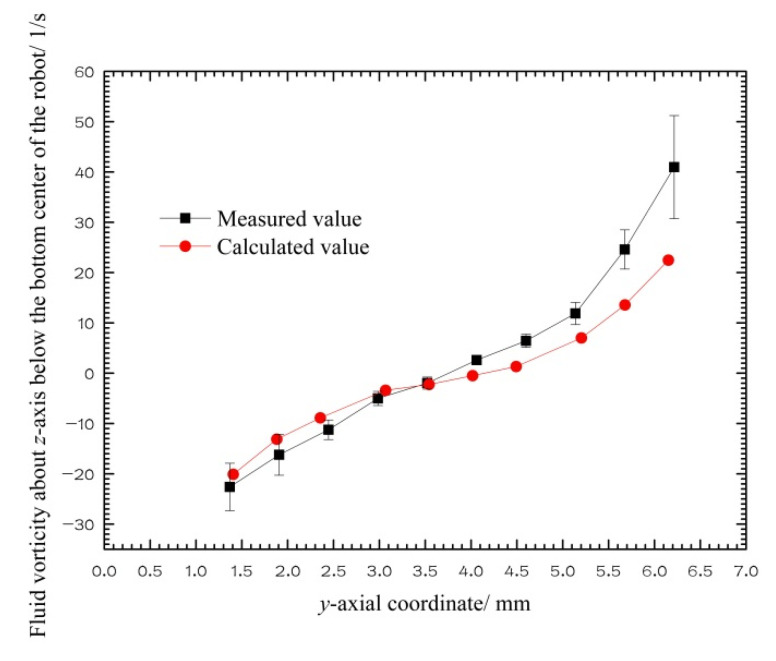
Fluid vorticity about the *z*-axis below the bottom center of the robot (*d* = 18 mm, *v* = 0.05 m/s, *n* = 60 r/min, *η* = 0.02 Pa·s).

Figure 12 is a comparison diagram of the CFD calculation and PIV measurement values of the fluid vorticity about the *z*-axis on the *y*-axis below the capsule robot in the coordinate system shown in Figure 10. From the bottom of the capsule robot down, the vorticity of the fluid about the *z*-axis is linearly changed from positive to negative; that is, the rotational direction of the fluid is changed, and the vorticity value first decreases and then increases. In particular, the numerical results of vorticity in the fluid region near the robot are very close to the experimental results. The agreement between the numerical results of velocity and vorticity and the experimental results proves that the CFD method used is feasible and accurate.

## 5. Performance Analysis of Capsule Robot in Complex Environment

### 5.1. Curved Pipe

**Figure 13:**
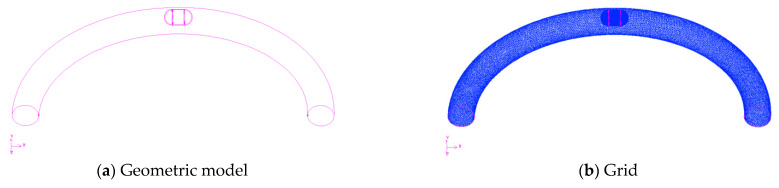
Capsule robot system in curved pipe (*d* = 18 mm).

In this section, we examine whether the operating performance of the capsule robot is affected when the pipe is bent. Figure 13 is a three-dimensional and grid diagram of the capsule robot system in a curved pipe. The inner diameter of the curved pipe is 18 mm, its radius of curvature is 100 mm, and the size of the capsule robot is the same as that in the straight pipe.

Table 1 shows the comparison of three performance indicators of the capsule robot for curved and straight pipes. The table shows that all performance indicators of the capsule robot in the curved pipe are similar to those in the straight pipe. Therefore, in the subsequent study, it was assumed in the numerical calculations that the capsule robot was in a straight pipe.

### 5.2. Peristaltic Environment

Intestinal peristalsis is a normal physiological phenomenon of the human body. It is a continuous and coordinated contraction of the circular and longitudinal muscles. It occurs at the proximal end of the small intestine and spreads to the distal end of the intestine. The spread rate is 0.5–2.0 cm per second, and the spread frequency is 8–11 times per minute.

In numerical calculations, as peristalsis of a pipe is more difficult to simulate, we assumed that the fluid in the pipe flows according to the spread rate and frequency of intestinal peristalsis to replace the pipe peristalsis. Assuming that the intestinal velocity is 2 cm/s at 9 times/min, the fluid velocity at the inlet of the pipe is as follows:

*u*(*t*) = 0.02sin0.942*t* (5)

where *t* is time.

Table 2 shows the performance comparison of active and passive capsules under the different fluid conditions of positive peristaltic flow, reverse peristaltic flow, and static flow. In the table, the time taken to calculate the results was the time of the maximum value of the peristaltic spread rate. The translational speed of the active capsule was 0.05 m/s, and the rotational speed was 60 r/min. The table shows that the positive peristaltic flow has little effect on the forward resistance of the capsule, but the reverse peristaltic flow increases the forward resistance of the capsule. The condition of positive or reverse peristaltic flow has little influence on the fluid turbulent intensity near the capsule; that is, it has little influence on the operating stability of the capsule. The maximum fluid pressure on the pipe wall is reduced by peristaltic flow, the effect of positive peristaltic flow is slightly reduced, and the effect of reverse peristaltic flow is significantly reduced. In the subsequent numerical calculation, the active capsule robot was used to precess in the fluid static flow.

## 6. Orthogonal Calculation and Analysis of Multi-Parameters of Capsule Robot

The orthogonal design method is an efficient, economic, and rapid experimental design method for multi-factor and multi-level problems. Compared with traditional experiment methods, it requires less time and has higher efficiency. As the orthogonal table has the advantages of “uniform dispersion and neat comparison”, the analysis of the orthogonal test results provides a comprehensive understanding of the test situation, and allows optimal test results to be obtained with as few tests as possible.

When the capsule robot precesses in a pipe filled with mucus, the operating performance indicators of the robot include: the forward resistance of the robot *F_r_*, which affects the passing capacity of the robot; the fluid turbulent intensity near the robot *I_t_*, which affects the operating stability of the robot; and the maximum fluid pressure on the pipe wall *P_m_*, which affects the degree of damage to the pipe. According to the three indicators, the influence of the relative degree, significance, and optimal combination of various parameters of the robot system were studied by orthogonal numerical calculation.

### 6.1. Orthogonal Numerical Calculation

Referring to Figure 1, four parameters which have a significant influence on the operating performance of capsule robot were selected as orthogonal design factors, namely, pipe diameter *d*, robotic translational speed *v*, robotic rotational speed *n*, and fluid viscosity *η*, which are represented by A, B, C, and D, respectively. Five levels are set for each factor, and the level value of each factor is more uniformly chosen in the reasonable operational condition of the intestinal capsule robot, thus forming an L_25_(5^4^) orthogonal table [28]. The four factors and five corresponding levels that affect the operating performance indicators of the capsule robot are shown in Table 3.

Due to the size of the calculation required for 625 (5^4^) numerical calculation schemes, 25 typical schemes were selected, as shown in Table 4. Each of the 25 groups of parameters of the robot system in Table 4 were modeled, meshed, and numerically calculated, and the corresponding operating performance indicators of the capsule robot were obtained.

### 6.2. Range Analysis of the Operating Performance Indicators of the Capsule Robot

Let *K_ij_* be the sum of the calculation indicators for the factor in column *j* and the level number *i*. *j* = *A* (*d*), *B* (*v*), *C* (*n*), and *D* (*η*); *i* = 1, 2, 3, 4, and 5.

(6) $$\bar{k_{ij}}=\frac{K_{ij}}{r_{ij}}$$

where $\bar{k_{ij}}$ is the average value of orthogonal calculation indicators for the factor in column *j* and the level number *i*; and *r_ij_* is the calculation time for the factor in column *j* and the level number *i*.

The range analysis method is used to analyze the influencing degree of each factor on the calculation indicators by comparing the range of each factor. The range *R_j_* is defined as the difference between the maximum value and the minimum value of the statistical parameter $\bar{k_{ij}}$ calculated at each level of the factor in column *j*.

(7) $$R_{j}=\max\{\bar{k_{ij}}\}-\min\{\bar{k_{ij}}\}$$

The range reflects the relative influence of each factor on the calculation indicator. The influencing degree of factors can be directly judged by *R*. The greater the range *R*, the greater the influence on the calculation indicator when the factor level is changed. Therefore, the factor with the largest range *R* is the main factor.

Through the range analysis of the three performance indicators of the capsule robot at the same level and the same factor in Table 4 (see Table 5), the relative influencing degree of various parameters on the operating performance of the capsule robot is reflected.

Table 5 shows that various parameters of the capsule robot system have different effects on the three performance indicators of the robot. Among these, the relative influencing degree of various factors on the forward resistance of the capsule robot *F_r_* is as follows: fluid viscosity > robotic translational speed > pipe diameter > robotic rotational speed; the relative influencing degree of various factors on the fluid turbulent intensity near the robot *I_t_* is as follows: fluid viscosity > robotic translational speed > robotic rotational speed > pipe diameter; and the relative influencing degree of various factors on the maximum fluid pressure to the pipe wall *P_m_* is as follows: fluid viscosity > pipe diameter > robotic translational speed > robotic rotational speed. It can be concluded that, during the precessing process of a capsule robot in a pipe filled with mucus, the changes in various parameters have different effects on each performance indicator of the capsule robot. Among these, the fluid viscosity has a greater impact on all performance indicators, whereas the robotic rotational speed has a smaller impact on all performance indicators.

### 6.3. Variance Analysis of the Operating Performance of the Capsule Robot

The order of importance among the influencing factors can be judged by the range analysis. However, to objectively judge the influencing significance of each factor on the calculation indicators, variance analysis is needed.

In variance analysis, the degree of variation of calculation data is composed of two parts. The first is the variation of calculation indicators caused by the change in each factor’s own level, which is represented by the squared sum of the group difference *S_j_*. The other is the variation caused by the calculation error. In the orthogonal design, the error is represented by setting a blank column. Therefore, the corresponding variance analysis is expressed by the squared sum of the group difference *S_e_* in the blank column.

(8) $$S_{j}=r\sum_{a=1}^{p}{\left({\bar{y}}_{aj}-\bar{y}\right)}^{2}$$

(9) $$S_{e}=\sum_{a=1}^{p}\sum_{b=1}^{r}{\left(y_{ab}-{\bar{y}}_{aj}\right)}^{2}$$

where *r* is the calculation time of each factor with the same level number; *p* is the level number of each factor; ${\bar{y}}_{aj}$ is the mean value of the calculation indicators for the *j*-th factor and level number *a*; $\bar{y}$ is the total mean value of the calculation indicators; and *y_ab_* is the calculation indicator value.

The basic idea of variance analysis is to calculate and compare the above two parts of variation, and the significance level of the corresponding factors on the calculation indicators is determined using a hypothesis test. Equation (8) shows that the larger the value of *S_j_*, the greater the influence of the factor level change on the indicator. The values of *S_j_* and *S_e_* are related to their own degrees of freedom *f_j_* and *f_e_*.

*f_j_* = *r_j_* – 1 (10)

*f_t_* = *n* – 1 (11)

(12) $$f_{e}=f_{t}-\sum f_{j}\;$$

where *f_j_* is the degrees of freedom of *S_j_*; *r_j_* is the calculation time of the *j*-th factor with the same level number; *f_t_* is the total degrees of freedom; *n* is total calculation time; *and f_e_* is the degree of freedom of *S_e_*.

After obtaining *S_j_* and *S_e_*, the hypothesis test is carried out to judge the significance of each influencing factor. For example, it is assumed that the fluctuation of factor *j* has a significant impact on the indicator. On the premise of this assumption, it is concluded that the statistic *F_j_*, which is related to *S_j_* and *S_e_*, obeys the *F* distribution:

(13) $$F_{j}=\frac{S_{j}/f_{j}}{S_{e}/f_{e}}∼F_{\alpha }(f_{j},f_{e})$$

where *α* is the significance level.

After calculating *F_j_*, the test *p*-value corresponding to the *F*-value can be obtained by looking up the *F* distribution table. Analysis of variance shows that: if *F_j_* > *F*_0_._01_, the factor is highly significant, and is expressed by “****”; if *F*_0_._01_ > *F_j_* > *F*_0_._05_, the factor is significant, and is expressed by “***”; if *F*_0_._05_ > *F_j_* > *F*_0_._1_, the factor has an influence, and is expressed by “**”; if *F*_0_._1_ > *F_j_* > *F*_0_._2_, the factor has a certain influence, and is expressed by “*”; and if *F_j_ < F*_0_._2_, the factor has no influence, and is expressed by “/”.

Table 6 shows the variance analysis of the three operating performance indicators of the capsule robot. The table shows that, for the performance indicator *F_r_*, the fluid viscosity has a certain influence, and the other three factors have almost no influence. For the performance indicator *I_t_*, the fluid viscosity has a highly significant influence, the robotic translational speed has a significant influence, the robotic rotational speed has a certain influence, and the pipe diameter has almost no influence. For the performance indicator *P_m_*, the fluid viscosity and the pipe diameter have a certain influence, and the other two factors have almost no influence. The above research shows that the fluid viscosity is an important factor that affects the passing capacity, stability, and non-invasion of the capsule robot; the translational speed of the robot plays an important role in its operating stability; and the pipe diameter is also an important factor that affects the damage of the pipe wall caused by the fluid pressure.

### 6.4. Parameter Optimization of the Capsule Robot System

According to the three performance indicators (*F_r_*, *I_t_*, and *P_m_*) of the capsule robot in the pipe filled with mucus, the parameters of the capsule robot system were optimized, and the parameters with the minimum level mean value of the performance indicators of the capsule robot were selected.

The diameters of the human intestinal tract are different in different positions, and they correspond to the different-diameter pipes in this paper. According to Table 5, for the pipes with different diameters, the optimal combination of various parameters of the capsule robot system with the minimum *F_r_* value as the optimization objective is B1C1D1; that is, the robotic translational speed is 0.02 m/s, the robotic rotational speed is 60 r/min, and the fluid viscosity is 0.005 Pa·s. Based on the numerical simulation with this set of optimized parameters, the forward resistance of the capsule robot *F_r_* is 0.86 mN, which is less than that obtained from the orthogonal table. The optimal combination of various parameters of the capsule robot system with the minimum *I_t_* value as the optimization objective is B2C1D1; that is, the robotic translational speed is 0.03 m/s, the robotic rotational speed is 60 r/min, and the fluid viscosity is 0.005 Pa·s. Based on the numerical simulation with this set of optimized parameters, the fluid turbulent intensity near the robot *I_t_* is 7.95%, which is close to the minimum value obtained from the orthogonal table. The optimal combination of the various parameters of the capsule robot system with the minimum *P_m_* value as the optimization objective is B1C1D1; that is, the robotic translational speed is 0.02 m/s, the robotic rotational speed is 60 r/min, and the fluid viscosity is 0.005 Pa·s. Based on the numerical simulation with this set of optimized parameters, the maximum fluid pressure on the pipe wall *P_m_* is 2.71 Pa, which is less than that obtained from the orthogonal table.

## 7. Conclusions

(1) A set of drive systems for a capsule robot in a pipe driven by an external permanent magnet, and a measurement system of the fluid flow field in the pipe during the robot’s precession, were designed and manufactured. The velocity of the fluid in the pipe of the capsule robot was calculated using the CFD method and measured using PIV technology. The numerical calculation values and experimental measurement values were similar, which verifies that the CFD method used in this paper is feasible and accurate. Furthermore, the performance of the capsule robots was numerically analyzed and compared in complex environments of a curved pipe and peristaltic flow;
(2) Range and variance analysis in orthogonal design was used to analyze the relative degree and significance of the influence of pipe diameter, robotic translational speed, robotic rotational speed, and fluid viscosity on the three performance indicators of the capsule robot. The fluid viscosity is an important factor that affects all the operating performance indicators of the capsule robot;
(3) Using the best passing capacity and operating stability of the capsule robot, and the minimum damage to the pipe, as the optimization objectives, the optimal combinations of various parameters of the capsule robot system were designed;
(4) Numerous factors affect the operating performance of the capsule robot, such as structural parameters of the robot, pipe characteristics, and magnetic field parameters. These parameters need to be studied. In addition, the interaction of various factors was not considered in this study. These issues will be studied in the future;
(5) The CFD method, PIV technology, and orthogonal design method used in this paper can be widely used in the calculation, measurement, and optimization of the fluid field of in-pipe capsule robots in a liquid environment.

## Figures and Tables

**Table 1:** Performance comparison of capsule robot in curved and straight pipes (*d* = 18 mm, *v* = 0.05 m/s, *n* = 60 r/min, and *η* = 0.02 Pa·s).

Environment and Difference	Forward Resistance of the Robot/ mN	Fluid Turbulent Intensity Near the Robot/%	Maximum Fluid Pressure to the Pipe Wall/Pa
Straight pipe	3.51	12.11	17.32
Curved pipe	3.62	12.34	17.13
Proportion of difference	3.13%	1.90%	1.1%

**Table 2:** Performance comparison of active and passive capsules in peristaltic flow and static flow (*d* = 18 mm, *v* = 0.05 m/s, *n* = 60 r/min, and *η* = 0.02 Pa·s).

Type	Fluid Flow State	Forward Resistance of the Robot/mN	Fluid Turbulent Intensity Near the Robot/%	Maximum Fluid Pressure to the Pipe Wall/Pa
Active capsule robot	Static flow	3.51	12.11	17.32
	Positive flow	3.50	12.14	3.89
	Reverse flow	5.27	12.83	−0.44
Passive capsule	Positive flow	−0.09	9.60	7.35
	Reverse flow	1.77	10.21	−0.33

**Table 3:** Levels and factors influencing the operating performance of the capsule robot.

Level	Factor			
	A (*d*)/mm	B (*v*)/m/s	C (*n*)/r/min	D (*η*)/Pa·s
1	14	0.02	60	0.005
2	16	0.03	90	0.01
3	18	0.04	120	0.02
4	20	0.05	150	0.05
5	22	0.06	180	0.1

**Table 4:** Orthogonal calculation values of the operating performance indicators of the capsule robot.

No.	A (*d*) /mm	B (*v*) /m/s	C (*n*) /r/min	D (*η*) /Pa·s	Factor Combination	Forward Resistance of the Robot *F_r_*/mN	Fluid Turbulent Intensity Near the Robot *I_t_*/%	Maximum Fluid Pressure to the Pipe Wall *P_m_*/Pa
1	14	0.02	60	0.005	A1B1C1D1	1.17	6.55	5.72
2	14	0.03	90	0.01	A1B2C2D2	2.57	8.21	15.39
3	14	0.04	120	0.02	A1B3C3D3	5.25	11.05	30.57
4	14	0.05	150	0.05	A1B4C4D4	13.17	17.08	78.94
5	14	0.06	180	0.1	A1B5C5D5	29.06	25.15	163.77
6	16	0.02	90	0.02	A2B1C2D3	1.58	9.60	7.35
7	16	0.03	120	0.05	A2B2C3D4	4.03	13.50	18.47
8	16	0.04	150	0.1	A2B3C4D5	8.81	19.32	42.31
9	16	0.05	180	0.005	A2B4C5D1	2.27	8.89	10.65
10	16	0.06	60	0.01	A2B5C1D2	3.72	10.30	20.47
11	18	0.02	120	0.1	A3B1C3D5	2.99	16.91	18.40
12	18	0.03	150	0.005	A3B2C4D1	1.24	9.40	4.54
13	18	0.04	180	0.01	A3B3C5D2	2.09	10.66	10.03
14	18	0.05	60	0.02	A3B4C1D3	3.51	12.11	17.32
15	18	0.06	90	0.05	A3B5C2D4	6.91	16.97	30.73
16	20	0.02	150	0.01	A4B1C4D2	1.01	11.01	3.53
17	20	0.03	180	0.02	A4B2C5D3	1.90	12.86	6.48
18	20	0.04	60	0.05	A4B3C1D4	3.57	13.81	14.18
19	20	0.05	90	0.1	A4B4C2D5	6.73	19.32	28.43
20	20	0.06	120	0.005	A4B5C3D1	2.93	12.73	8.45
21	22	0.02	180	0.05	A5B1C5D4	1.65	15.90	18.00
22	22	0.03	60	0.1	A5B2C1D5	3.26	15.63	19.59
23	22	0.04	90	0.005	A5B3C2D1	1.74	10.10	5.66
24	22	0.05	120	0.01	A5B4C3D2	2.76	12.18	9.61
25	22	0.06	150	0.02	A5B5C4D3	4.45	15.32	17.88

**Table 5:** Range analysis of the operating performance indicators of the capsule robot.

Performance Indicator		Influencing Factor			
		*d*/mm	*v*/m/s	*n*/r/min	*η*/Pa·s
*F_r_* /mN	Level 1	10.24	1.68	3.04	1.87
	Level 2	4.08	2.60	3.91	2.43
	Level 3	3.35	4.29	3.59	3.34
	Level 4	3.23	5.69	5.74	5.86
	Level 5	2.77	9.41	7.39	10.17
	Mean range	7.47	7.73	4.35	8.30
*I_t_* /%	Level 1	13.61	11.99	11.68	9.53
	Level 2	12.32	11.92	12.84	10.47
	Level 3	13.21	12.99	13.27	12.19
	Level 4	13.95	13.92	14.43	15.45
	Level 5	13.83	16.09	14.69	19.27
	Mean range	1.62	4.17	3.01	9.73
*P_m_* /Pa	Level 1	58.88	10.60	15.46	7.00
	Level 2	19.85	12.89	17.51	11.81
	Level 3	16.20	20.55	17.10	15.92
	Level 4	12.21	28.99	29.44	32.06
	Level 5	14.15	48.26	41.79	54.50
	Mean range	46.66	37.66	26.33	47.50

**Table 6:** Variance analysis of the operating performance indicators of the capsule robot.

Performance Indicator	Source of Variance	Square Sum	Degree of Freedom	Mean Square	*F* Value	*F_α_*	Significance
*F_r_*	*d*	194.10	4	48.53	1.57	*F*_0_._2_(4,20) = 1.7 *F*_0_._1_(4,20) = 2.25 *F*_0_._05_(4,20) = 2.87 *F*_0_._01_(4,20) = 4.43	/
	*v*	184.42	4	46.11	1.49		/
	*n*	64.65	4	16.16	0.52		/
	*η*	231.33	4	57.83	1.87		*
	*e*	123.72	4	30.93			
*I_t_*	*d*	8.59	4	2.15	0.63		/
	*v*	59.20	4	14.80	4.34		***
	*n*	30.07	4	7.52	2.21		*
	*η*	318.06	4	79.52	23.33		****
	*e*	13.63	4	3.41			
*P_m_*	*d*	7650.56	4	1912.64	1.95		*
	*v*	4639.59	4	1159.90	1.18		/
	*n*	2541.52	4	635.38	0.65		/
	*η*	7488.94	4	1872.23	1.90		*
	*e*	3932.26	4	983.06			

## Data Availability

The study did not report any data.
